# Supplementary material for: Return of individual research results from genomic research: A systematic review of stakeholder perspectives
Source: PLoS One. 2021 Nov 8;16(11):e0258646. doi: 10.1371/journal.pone.0258646 (PMC8575249; doi:10.1371/journal.pone.0258646)
Supplement: S1 Table — (PDF) [file pone.0258646.s001.pdf]

Study Overview

**Your name:**	
**Date (DD MM YYYY):**	

**Alert(s) to Review Team leads:**		Free text space to communicate important information to Review Team leads (e.g. paper does not appear to fit review criteria; PDF is incorrect version of paper).

**Study aims:**		Cut-and-paste aim of study directly from paper (or summarise if simpler).
**Year(s) of data collection:**		Put questions marks after years if uncertain/unclear. Use 'Not stated' if information not provided.

**Type of research:**	Drop down menu	Quantitative<br>Qualitative<br>Mixed methods
**Specific method(s):**	Drop down menu	Survey (including questionnaires)<br>Interviews<br>Focus groups<br>Survey + Interviews<br>Survey + Focus groups<br>Interviews + Focus groups<br>Other (please state)
**Setting(s):**		Briefly describe setting(s) where data were collected (e.g. online survey; telephone survey; face-to-face telephone interviews; focus groups in communities).

**Format:**	Drop down menu	Return of existing data / Direct experience<br>Return of hypothetical data / Hypothetical scenario(s)<br>Unclear / Not stated<br>Other (please specify)

**Country:**		Indicate specific country. If multinational, indicate all countries involved. If necessary, write 'Not stated' and indicate most probable country/ies.

Stakeholders

**Stakeholders represented:**		Describe who participated in study. Include details if provided (e.g. genetic counsellors; general public; family members of deceased men who had participated in study to identify genetic contributions to early-onset prostate cancer related to BRCA2).
**Inclusion / exclusion criteria:**		Provide details about who was and want not invited in study. Use 'Not stated' as necessary.

**Stakeholder characteristics:**		Describe stakeholders, especially if not White, middle class, educated and from higher income Western country/ies.
**Age group(s):**	Drop down menu	Adults (≥age 17)<br>Children (<age 18)<br>Adults + Children<br>Other (please state)
**Sample size:**		Indicate as n=x. Provide breakdown where appropriate (e.g. Survey n=145 + Interviews n=14). Use cell on right for additional information as necessary.

**Sampling procedure:**		Indicate how participants were sampled. Differentiate between qualitative and quantitative methods as necessary.
**Recruitment method:**		Describe all methods used to contact participants for recruitment into study. Differentiate between qualitative and quantitative study arms as necessary.

Quantitative Results		
**Method of analysis:**		Note any particular method of analysis used to frame study and analyse data. Use 'Unclear / Not stated' as necessary.
**Key findings:**		Identify key findings related to research question, “What are stakeholders’ views and experiences regarding return of individual results from genomic research?”. Cut-and-paste from paper where possible.
**Author explanations:**		How do authors explain their findings? Cut-and-paste from paper as necessary. Use 'Not stated' as necessary.

Qualitative Results		
**Method of analysis:**		Note any theoretical framework or method of analysis used to frame study and analyse data (e.g. thematic analysis, interpretive phenomenological analysis). Use 'Unclear / Not stated' plus details as necessary.
**Key findings:**		Identify key findings related to research question, “What are stakeholders’ views and experiences regarding return of individual results from genomic research?”. Cut-and-paste from paper where possible.
**Author explanations:**		How do authors explain their findings? Cut-and-paste from paper as necessary. Use 'Not stated' as necessary.

Conclusions		
**Conclusions:**		What main conclusions are drawn? Cut-and-paste from paper where possible.
**Recommendations / Implications:**		Note any recommendations or implications for practice put forward by authors. Cut-and-paste from paper where possible.
**Study strengths:**		Any strengths identified by authors or by you as reviewer (please distinguish yours from theirs). Cut-and-paste from paper where possible.
**Study limitations:**		Any limitations identified by authors or by you as reviewer (please distinguish yours from theirs). Cut-and-paste from paper where possible.

Before you go…		
**Reviewer's notes / comments:**		Space for any final thoughts or for feedback to Review Team leads about this paper in particular or review process in general.
**References to follow-up:**		Please cut-and-paste any reference(s) from paper under review or elsewhere that meet study inclusion criteria. Complete list of papers identified for inclusion in review is in OneDrive folder alongside PDFs.
