# Supplementary material for: Return of individual research results from genomic research: A systematic review of stakeholder perspectives
Source: PLoS One. 2021 Nov 8;16(11):e0258646. doi: 10.1371/journal.pone.0258646 (PMC8575249; doi:10.1371/journal.pone.0258646)
Supplement: S2 Table — (PDF) [file pone.0258646.s002.pdf]

Theme	Sub-theme	Syntax Groupings
**Any term/phrase from Theme 1…**		
**Genetic data of interest**	Usefulness	(clinical OR personal) utility
		actionable
		(genomic OR genetic) result*
	Singularity	individual /n finding*
		individual /n result*
	Significance	variant* /n significance
		pertinent /n (result* OR finding*)
	Sharing	return* /n (result* or finding*)
		recei* /n (result* or finding*)
		disclos*
	Unexpectedness	(incidental OR secondary OR additional OR extraneous OR unsolicited OR ancillary) /n finding*
**AND any term/phrase from Theme 2…**		
**Genes**	Genes	"clinical sequenc*" OR gwas OR exom* OR genom* OR genet*
**AND any term/phrase from Theme 3**		
**Empirical context**	Point of view	view* OR opinion* OR experience* OR attitude* OR perspective* OR perception* OR preference* OR choice*
	Qualitative method	"focus group*" OR interview* OR survey* OR questionnaire*
