# Supplementary material for: Return of individual research results from genomic research: A systematic review of stakeholder perspectives
Source: PLoS One. 2021 Nov 8;16(11):e0258646. doi: 10.1371/journal.pone.0258646 (PMC8575249; doi:10.1371/journal.pone.0258646)
Supplement: Attachment — Submitted filename: Response to Reviewers.docx [file pone.0258646.s004.docx]

**Response to reviewers**

**Reviewers' comments:**
***Reviewer #3***

1. Table 2 is very well summarised, but I considered the full citation is not needed, the abbreviated citation will be enough. In that way the table can be shortened.

*While we thank the reviewer for their suggestion and agree that a shortened table would, in some ways, be desirable, in our opinion the context that is provided to the table by including the full reference (particularly the title) provides the reader with a valuable resource. As such, we believe it is in the readers’ interests that we leave it as it currently stands.*

2. The idea in line 256-257 is not clear

*We thank the reviewer for picking up this error. The sentence now reads “We used content analysis to enable systematic analysis of the methodologically diverse articles in this review.”*

3. Check the verb in line 710

*We thank the reviewer for picking up this error. The sentence now reads “Researchers also suggested returning results shows respect for patient-participant autonomy and recognizes participants’ ownership of their data.”*

4. Label heading and subheadings with numbers to make a easy reading pattern

*Headings and subheadings in the results section have been numbered.*

5. In lines 886-887 number are written and number as used as symbols better to be consistent through the text

We thank the reviewer for their suggestion. However, throughout the text we have consistently used text for numbers 1-9 and symbols for numbers 10 and above. We understand this to be an accepted way to report numbers.

6. In line 1117 the word “well-being” is separated by a high fen but not in Table 6 and other parts in the text

*We thank the reviewer for picking up this error. We have removed the hyphen in this instance and double-checked the remaining text.*

7. Some sentences must be split or they need to have more punctuation marks

*We have tried to split long sentences or add more punctuation throughout.*

While revising your submission, please upload your figure files to the Preflight Analysis and Conversion Engine (PACE) digital diagnostic tool, <https://pacev2.apexcovantage.com/>. PACE helps ensure that figures meet PLOS requirements. To use PACE, you must first register as a user. Registration is free. Then, login and navigate to the UPLOAD tab, where you will find detailed instructions on how to use the tool. If you encounter any issues or have any questions when using PACE, please email PLOS at [figures@plos.org](mailto:figures@plos.org). Please note that Supporting Information files do not need this step.

*All images were checked on the previous submission and, as we have not changed any of them, we have not rechecked the figures.*

Thank you for your ongoing consideration of our manuscript.

Best wishes,

Danya Vears

Murdoch Children’s Research Institute
